# Longitudinal Transcriptomic Analysis Reveals Systemic Effects of Risdiplam in Adults with Spinal Muscular Atrophy

**DOI:** 10.3390/brainsci16060643

**Published:** 2026-06-17

**Authors:** Maria Liguori, Arianna Consiglio, Eustachio D’Errico, Ylenia Antonacci, Martina Coffa, Alessandro Introna, Isabella Laura Simone

**Affiliations:** 1 National Research Council, Department of Biomedicine, Institute of Biomedical Technologies, Bari Unit, 70125 Bari, Italy; arianna.consiglio@cnr.it (A.C.); ylenia.antonacci@cnr.it (Y.A.); martinacoffa01@gmail.com (M.C.); 2 Neurology Unit, Department of Translational Biomedicine and Neuroscience, University of Bari “Aldo Moro”, 70124 Bari, Italy; eustachio.derrico@policlinico.ba.it (E.D.); ale.introna@gmail.com (A.I.); 3 National Research Council, Institute of Biomembranes, Bioenergetics and Molecular Biotechnologies, 70125 Bari, Italy; 4 School of Medicine, University of Bari “Aldo Moro”, 70124 Bari, Italy; isimone51@gmail.com

**Keywords:** spinal muscular atrophy, Risdiplam, Transcriptomics, *SMN2* gene, RNA sequencing

## Abstract

**Highlights:**

**What are the main findings?**

• In our SMA cohort, an increased number of full-length SMN2 transcripts was found under Risdiplam;
• Before treatment, genetic differences between SMA type II and type III reveal molecular distinctions between SMA phenotypes.

**What are the implications of the main findings?**

• Although exploratory, these findings support the efficacy of Risdiplam in adult SMA;
• The differential expression of several genes may impact some clinical features, i.e., different prognosis or response to therapies.

**Abstract:**

Background: Spinal Muscular Atrophy (SMA) is a neurodegenerative disease caused by reduced survival motor neuron (SMN) protein levels due to *SMN1* gene mutations. The natural history of SMA has dramatically changed since innovative therapies were approved; among them, Risdiplam (an oral molecule) increases the peripheral levels of SMN by modifying the pre-mRNA slicing of the paralogous *SMN2* that also codes for the protein. Methods: We performed longitudinal RNA sequencing on peripheral blood samples from 16 adult SMA patients (types II and III) before and after 12 months of Risdiplam treatment to assess transcriptomic changes. Results: During Risdiplam treatment, increased *SMN2* transcript levels were observed, which was coherent with the clinical condition of the investigated SMA cohort. Upregulated mitochondria genes or pseudogenes (i.e., *MT-ATP8* and *MTND1P11*) and downregulated autophagy-related pathways were also found. Baseline differences in gene expression between SMA type II and type III involved neurodegenerative (i.e., *MS4A3*, *C4BPA*, and *NEILS3*) and immune-related (*B2M*) genes. Conclusions: These findings support Risdiplam’s systemic impact in adult SMA subjects and reveal molecular distinctions between SMA phenotypes (types II and III), which may be of some relevance for future clinical and therapeutic strategies.

## 1. Introduction

Spinal Muscular Atrophy (SMA) is a rare neurodegenerative disease caused by biallelic mutations in the survival motor neuron-1 gene (*SMN1*) that lead to reduced expression of the corresponding protein SMN [1,2]. Indeed, up to 10% of the total SMN is produced by a paralogous gene “*SMN2*”, which is nearly identical to “*SMN1*” except for five nucleotide differences. One of these variations disrupts an exonic splicer enhancer and creates a splicing silencer in exon 7. As a result, approximately 90% of *SMN2*-derived transcripts lack exon 7, producing a truncated or unstable SMN protein [3,4,5]. However, the number of *SMN2* gene copies varies among individuals with SMA and significantly influences clinical prognosis. Disease phenotypes are classified as SMA type 0–IV based on the age at onset and disability scores, with the lower types representing a more severe form associated with very low SMN production [6,7].

SMN is a ubiquitous protein that interacts with more than 60 other proteins [8]. When SMN levels fall below 10–20% of normal, as seen in SMA types 0 and I, the disease manifests in severe or lethal forms. In contrast, individuals with SMA types II–IV may survive into adulthood, but experience varying degrees of disability that are inversely correlated with the SMN protein levels and the number of *SMN2* gene copies [9]. Similar findings have been observed in SMA animal models, supporting the role of *SMN2* as a modifier gene in SMA [10,11].

The natural history of SMA has dramatically changed in recent years with the approval of three innovative and effective therapies. These include an *SMN2*-targeting antisense oligonucleotide (Nusinersen) [12] and a small molecule (Risdiplam) [13], both of which modify *SMN2* pre-messenger RNA splicing and are approved for use in both children and adults with SMA. Additionally, an *SMN1* replacement therapy using an adeno-associated virus vector (Onasemnogene abeparvovec) [14] is available for patients younger than 2 years of age. While clinical improvements with these therapies are often significant in young SMA patients, the impact in adults is generally less pronounced and is frequently limited to the stabilization of disability rather than substantial improvement [15].

Before the authorization of Risdiplam, the only available therapy for adults with SMA was Nusinersen; however, its intrathecal administration was often painful or not feasible due to severe scoliosis or other skeletal malformation [16,17]. In contrast, Risdiplam is a systemically distributed small molecule administered orally once a day, representing a significant advancement in the treatment of adult SMA. Several real-world studies have confirmed its efficacy and safety [18,19,20,21]. As a mechanism of action, Risdiplam targets the genetic cause of SMA by promoting the inclusion of exon 7 in *SMN2* pre-mRNA splicing, thereby increasing the production of the functional SMN protein to compensate for the loss of *SMN1* function [22].

In this study, we investigated the molecular effect of Risdiplam in the systemic compartment, specifically peripheral blood, using a 12-month longitudinal RNA-seq analysis in adult SMA subjects. Additionally, we explored other transcriptomic differences that may characterize the phenotypes within our SMA cohort, with the aim of providing further insights into this degenerative neuromuscular disorder.

## 2. Materials and Methods

### 2.1. Subject Recruitment and Clinical Evaluation

The study was conducted in accordance with the Declaration of Helsinki, and approved by the local Ethical Committee of Azienda Ospedaliero-Unversitaria “Consorziale Policlinico” in Bari (protocol no. 0053567/16/96/2021). 

Adult patients (aged ≥ 18 years) with SMA types II–III [23] were recruited at the Neurology Unit of the Department of Translational Biomedicine and Neuroscience, University of Bari (Bari, Italy). Genetic tests identifying 5q SMA homozygous gene deletion, homozygous mutation, or compound heterozygotes confirmed the SMA diagnosis, with the number of *SMN2* copies ≤ 4. According to the drug protocol, they received a 5 mg dose once daily; for patients who were previously under Nusinersen treatment, a 6-month wash-out period was observed.

Patients were evaluated by experienced neurologists, and their clinical disabilities were measured using the Revised Upper Limb Module (RULM) and Hammersmith Functional Motor Scale (HFMS) [24,25]. Recruited patients had the ability to understand and comply with the study, as well as the ability to give written informed consent.

Exclusion Criteria: Patients with motor neuron diseases other than genetic SMA were excluded, as well as those with preexisting conditions such as HIV, clinically significant chronic hepatitis, or other active infections. Also, female patients confirmed to be pregnant (by laboratory testing) or lactating were not considered candidates for the therapy.

### 2.2. Molecular Analysis

#### 2.2.1. Sample Preparation

Peripheral blood samples were collected from patients at T0 and T12 and stored at −20 °C in 3 mL PAXgene Blood RNA Tubes (PreAnalytiX Qiagen/BD, Hombrechtikon, Switzerland) until use. Total RNA was isolated using the PAXgene Blood RNA Kit (PreAnalytiX Qiagen/BD, Hilden, Germany) at ITB CNR, Bari Unit. RNA concentration and purity were measured by Nanodrop ND-1000 (Thermo Scientific, Wilmington, DE, USA) and RNA 6000 Pico chip on the Bioanalyzer 2100 (Agilent Technologies, Santa Clara, CA, USA), respectively. Samples with RNA Integrity Number (RIN) scores higher than 7 and with A260/A280 values in the 1.8–2.2 range were processed in downstream deep sequencing.

#### 2.2.2. Quality Control

mRNA libraries were prepared with Illumina Stranded Total RNA Prep kit, ligation with RIBO zero PLUS. The quality of libraries was confirmed on the Bioanalyzer 2100 using the (Agilent Technologies, Santa Clara, CA, USA). The libraries were then pooled equimolarly into a multiplex sequencing pool and sequenced on a NovaSeq6000 sequencing platform (San Diego, CA, USA) to generate 2 × 100 bp paired-end reads, generating a final output of around 100 million reads per sample. Details of the workflow are reported elsewhere [26].

### 2.3. Bioinformatic and Biostatistical Analyses

After checking the quality of FASTQ files with FastQC and MultiQC [27], RNA-seq reads were mapped on Ensembl 114 with STAR [28] and RSEM [29]. Read counts were computed with RSEM estimation and evaluated with MultiDEA [30] to annotate gene similarities that cause mapping uncertainty. Differential expression analysis was performed with DESeq2 [31]. The results of differential expression analysis were considered statistically significant with an FDR ≤ 0.05 (PPDE > 0.95), with a mean read count > 25, and an absolute log_2_ fold change > 0.585 (corresponding to an absolute FC > 1.5). We applied these thresholds when comparing SMA type II vs. type III, T0 vs. T12 in paired mode (7 samples) and globally (16 vs. 7 samples).

MultiDEA was also applied to report the ambiguity in the *SMN1* and *SMN2* mapping, highlighting that almost 100% of the reads that mapped to one of these two genes also mapped identically to the other, while there was no ambiguity in these reads compared to other genes (no other genes showed significant portions in common with *SMN1* and *SMN2*). To evaluate the effect of the therapy on the isoform level, we counted how many reads covered the junctions between exons 6, 7, and 8 to look for an increase in the inclusion of exon 7 in patients.

### 2.4. Functional and Network Analyses

To capture all potentially relevant expression changes in the functional and network analyses, genes for the network analysis were selected based on an (absolute) FC ≥ 1.2 criterion. We included only genes upregulated in ≥4 subjects (and downregulated in none) and genes downregulated in ≥4 subjects (and upregulated in none).

Functional enrichment was performed using the g:Profiler platform [32], with Homo sapiens as the reference organism and Ensembl Gene IDs as identifiers. Terms derived from Gene Ontology (GO: Biological Process, Molecular Function, and Cellular Component), KEGG, and Reactome were considered, applying the g:SCS multiple testing correction method (default setting), and categories with *p* < 0.05 were regarded as significant. Enrichment analysis was applied using the DAVID 2021 platform [33,34], querying most of the available categories, including Gene Ontology (GOTERM_BP_DIRECT, GOTERM_MF_DIRECT, GOTERM_CC_DIRECT), metabolic and signaling pathways (KEGG_PATHWAY, BIOCARTA), protein domains (INTERPRO, SMART), UniProt annotations (UP_KW_BIOLOGICAL_PROCESS, UP_KW_CELLULAR_COMPONENT, etc.), and disease annotations (OMIM_DISEASE). Results were analyzed considering functional annotation clusters, ranked according to the Enrichment Score provided by the software. Clusters with an Enrichment Score ≥ 1.3 were considered, and the most representative biological terms were selected within each cluster.

Transcriptional regulatory networks were reconstructed in Cytoscape v3.10.3 [35] starting from a table of transcription factor (TF)–gene interactions, formatted with the columns: TF, Gene, Interaction, and Score (−log10 *p*-adjusted). A second table containing node metadata (ID, type, and associated pathway) was imported as an attribute table. The network was generated by defining transcription factors as sources and genes as targets. Visualization was supported by graphical attributes: nodes were colored according to their associated pathway, and interactions were scaled based on the TF–gene binding score. 

## 3. Results

### 3.1. Overall SMA Population

Sixteen patients with adult SMA (10 females, 6 males; mean age: 37.6 ± 10.8 years) were recruited for this observation; based on their age at onset, they were classified as SMA type II (no. 11) and SMA type III (no. 5). Individual details of their demographic and clinical features are summarized in Table 1.

None of the subjects discontinued Risdiplam intake during the 12-month interval of this molecular observation, after which only one subject (SMA-15) decided to withdraw from the therapy due to severe gastrointestinal side effects. Clinical data of SMA-26 were not available since she participated in a clinical trial (data are protected to preserve double-blinding).

We were able to perform an RNA-seq analysis at the time of enrollment (before the first Risdiplam administration, T0) and after 12 months’ treatment (T12) in seven patients.

### 3.2. Risdiplam T0 vs. T12

The overall analysis of DE genes between SMA patients at T0 (no. 16) and at T12 (no. 7) revealed a statistically significant increased expression of mitochondrial genes or pseudogenes (*MT-ATP8*, *MTND1P11*, *MTND5P14*, *MT-ATP6*, *MT-ND5*, *MT-ND4*, and *MT-ND6*), while the only decreased gene is the immunoglobulin *IGKV1D-39* (see Table 2).

When the longitudinal data T0–T12 were individually paired (i.e., within-subject changes), we found that the expression of 15 genetic regions resulted significantly increased and 2 decreased at T12 compared to the baseline status; most of these regions were novel transcripts, the remaining were genes (*CAND2* and *SNHG31*), long non-coding RNAs (*TRIM8-DT*), an intronic transcript (*ACAP2-IT1*), and pseudogenes (*MT-ATP8*, *MTND1P11*, *MTCO2P12*, and *CHORDC1P4*) whose functional meanings need to be explored (Table S1).

Furthermore, looking at the impact of the therapy on the *SMN2* copies (exons 6–7–8 boundaries) (Table 3), the data showed in all subjects an increase in at least one of the regions containing exon 7 (FC > 1.5), especially the exon 7–8 junction, although this was without statistical relevance; no further speculations are allowed due to the small counts found, which were possibly due to the early investigation, although this trend seemed to be consistent with the clinical conditions of the selected subjects. Subjects SMA-14 and SMA-22 showed a discrete clinical improvement, at least in the RULM score, while SMA-17 and SMA-20 were substantially stable (Table 1).

All the sequenced genetic regions are reported in Table S2.

#### Pathways/Network Analyses

The functional annotation clustering analysis (DAVID) performed in the therapy interval identified several clusters composed of genes potentially involved in SMA-related molecular processes. In particular, the overexpressed genes (289 genes; Table S2) were mainly enriched in biological processes related to ubiquitin conjugation (GO:0016567), RNA splicing (GO:0005515), and protein binding (GO:0008380). These enrichments suggest an involvement of post-translational modification systems and RNA-processing mechanisms during Risdiplam treatment.

On the other hand, the underexpressed genes (248 genes; Table S2) showed enrichment in pathways associated with autophagy (GO:0006914) and cell–cell communication, including annotations such as signal (KW-0732), glycoprotein (KW-0325), membrane (KW-0472), and transmembrane (KW-0812). These preliminary findings indicate dysfunction in intracellular degradation pathways and intercellular signaling components. Downregulated genes at T12 were of particular interest. The g:Profiler analysis showed that a substantial proportion of these genes were enriched as targets of Transcription Factors (TFs), including: *TFAP2A*, known to regulate proliferation and cellular differentiation; *E2F*, a key regulator of the cellular cycle; and the ubiquitous *SP1* (Figure S1).

**Figure 1:**
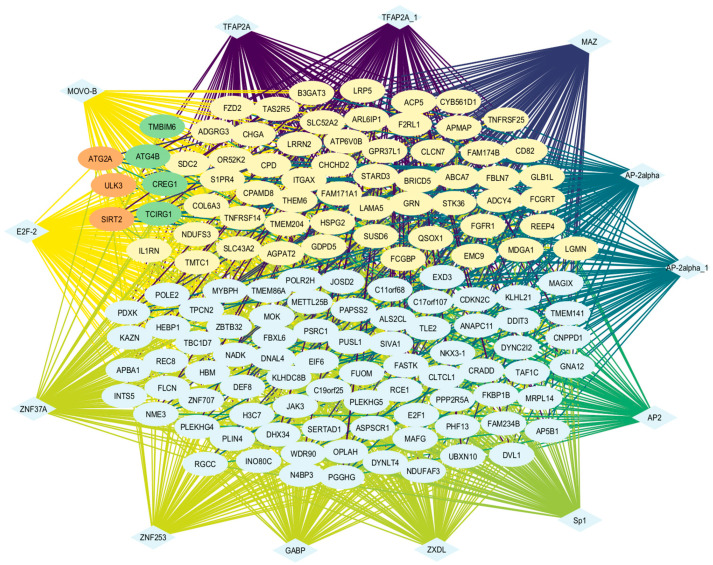
Graphical representation of computationally predicted mRNA–TF interactions (genes underexpressed at T12). Legend: TFs are diamonds, targeted genes are ovals; orange nodes represent autophagy defects, yellow nodes indicate lysosomal defects, green nodes stand for both, light blue for the remaining. The color intensity of the connecting lines represents the strength of the interaction—the darker it is, the stronger the interaction (violet: ~4.33, yellow: ~2.89) (by Cytoscape v3.10.3). TFs = transcription factors.

Overall, these findings point to a complex molecular framework involving TFs and their downstream target genes, with a potential link to pathways related to autophagy and lysosomal function during the T0–T12 Risdiplam interval (Figure 1).

### 3.3. SMA Type II vs. Type III

At baseline, the molecular analysis of DE genes revealed a statistically distinct molecular profile between SMA type II and type III patients, with all identified DE genes showing reduced expression in the latter group (Table 4).

Notably, most of these genes (13/19) have already been reported in association with neurodegenerative processes, including dementias. Pathway enrichment analysis (DAVID) showed that they are mainly associated with cytoplasmic, cytosolic, and ribosomal components (GO:0005737, GO:0005829, and GO:0005840, respectively), and are functionally implicated in RNA binding (GO:0003723).

Given the small subgroup size, these findings should be interpreted with caution; however, they raise the possibility that alterations in cellular components and processes involved in RNA metabolism and protein synthesis may help discriminate between SMA type II and type III patients.

## 4. Discussion

Since SMN is a ubiquitous protein and Risdiplam is a small molecule with an excellent body-wide distribution [54,55], the transcriptomic-wide effect of Risdiplam has previously been explored in cultured fibroblasts belonging to a SMA type I patient. The most suggestive results showed changes in the expression of genes associated with DNA replication, cell cycle, RNA metabolism, cell signaling, and metabolic pathways [56].

Here, we investigated the possible impact of Risdiplam on circulating (PBMC) mRNAs. As in the previous investigation on Nusinersen [57], under Risdiplam we found an increased number of full-length SMN2 transcripts, which was coherent with the clinical conditions of the selected cohort. In our view, this was particularly interesting considering the oral route of administration and the systemic distribution of the molecule. This evidence, together with the overall tolerability of the drug (at least in our study sample) and the stable or improved clinical conditions of the SMA patients, further supports the use of Risdiplam in the adult SMA population. As detailed in Tables S3 and S4, SMA modifier genes—such as *NCALD, CORO1C*, and *HNRNPA1* [58]—or genes belonging to the SMN complex—like *Gemin2-8* and *STRAP* [59]—did not significantly change in both the paired and overall comparisons, so we may speculate that they did not impact SMN2 transcript levels (at baseline and T12), thus altering the observed molecular response of Risdiplam.

Other findings with possible clinical effect derived from the significant overexpression at T12 of genes related to mitochondrial structure and function, like *MT-ATP6/8*—both implicated in ATPase activity—and *MT-ND4/5/6*—related, i.e., to defects in oxidative phosphorylation (OXPHOS)—which characterize diseases like LHON, MELAS, and MERRF [60,61].

Indeed, the involvement of mitochondria in SMA pathogenesis via the ubiquitous SMN protein has been deeply explored and confirmed, especially in the last few years. Among these, an interesting transcriptomic analysis on skeletal muscle biopsies of SMA type II patients showed an enrichment of mitochondrial processes—such as oxidative phosphorylation (OXPHOS) and citric acid cycle (TCA)—among the downregulated genes, whereas an enrichment of calcium signaling and p53 target pathways was found for upregulated genes. The mtDNA copy number from extracted DNA in muscle, as in PBMC, also revealed a significant decrease in both SMA tissues, although further evaluations demonstrated that this change is a consequence of muscle damage rather than an intrinsic feature of SMA [62]. Of interest, for some of these patients, Nusinersen therapy was noted in the clinical data collected, although the duration of treatment at the sampling time was not recorded, as well as no pre-treatment data being available.

In our study, molecular data referred to as T0 represent the naïve condition of SMA subjects (i.e., before any targeted therapy), so the mitochondrial status change looks plausible; therefore, we might hypothesize that Risdiplam also acted peripherally via increased mitochondrial activity measured at the PBMC level, which might impact upon clinical disabilities. Whether this change reflects a compensatory response under therapy rather than true mitochondrial overactivity remains speculative. Furthermore, this analysis did not consider all relevant variables, e.g., the different leukocyte composition of the samples, and so conclusions should be postponed until further in-depth evaluation.

Further interesting results during Risdiplam treatment came from the evaluation of the overall pathogenic pathways, i.e., after 12 months (T12); genes implicated in ubiquitin conjugation, like *SUMO* (small ubiquitin-like modifier), were found to be overexpressed (e.g., *HTATSF1, XPA*, and *ZNF618*), which is particularly compelling since Sumoylation is important for the proper assembly and function of the SMN complex. In this direction, an experimental animal study of SMA showed that loss of post-translational modification compromises the ability to rescue motor axon deficits [63]. Similarly, the expression of genes (e.g., *FAM184A, EPB41L2*, and *SQLE*) that codify for proteins involved in mRNA binding and processing within the cellular nuclei was found to be overexpressed. Functional studies are needed to confirm the suggestion that these genes may act with beneficial effect when stimulated by Risdiplam.

The pathway analysis showed at T12 the significant downregulation of genes participating in the autophagy network, like *ATG2A*, *ULK3*, and *SIRT2* (see Figure 1). Several lines of experimental evidence have highlighted the dysfunctional autophagosomal activity observed in both motor neuron soma and neuritis in severe SMA mice, which confirms the detrimental role of impaired or excessive autophagy, i.e., for the accumulation of protein aggregates instead of its rather protective role to promote survival [64,65]. We also observed a significant downregulation of genes involved in lysosomal activities, i.e., *LGMN*, *LRP5*, *LRRN2*, *COL6A3*, *NDUFS3*, *GRN*, and *STARD3*, and a consistent number of others affecting both activities, like *TMBIM6*, *ATG4B*, *CREG1*, and *TCIRG1* (Figure 1). Both lysosomal dysfunctions [66] and autophagy stalling [67] have been reported in neurodegeneration, but how they interact in the pathogenic complexity of SMA or whether they may be conditioned by small-molecule drugs like Risdiplam is difficult to conclude without functional validation. It is, however, noteworthy that our data are, so far, the first derived from a longitudinal early observation under SMN-targeted therapy; although preliminary, we hope that they can give educated suggestions for further therapeutic efforts.

Despite the huge progress in understanding the pathogenesis of neurodegenerative diseases, the definition of the whole picture is still challenging, even in cases of disorders like SMA, where the genetic cause is known. Among the others, the molecular differences underlying the disease phenotypes still need to be disentangled, which is crucial, i.e., to identify key disease targets for future treatments, and to verify whether these discrepancies may justify events like the different responses to already available therapies, or the onset of side effects.

One of the key points may lie in the phenotypic variability that characterizes SMA, starting from the classification by type; in fact, beyond age at disease onset and motor abilities, we were wondering whether a peculiar “genetic background” would be pictured, i.e., in our study population of SMA types II/III. Of interest, our data showed that most of the differentially expressed genes between the two groups were implicated in neurodegenerative processes—as reported in other NDDs (see Table 4)—or involved in immunological mechanisms that were T-cell-mediated (*B2M*) [49] or in nerve functioning (*SF3B6* and *TPT1*) [37,40]. The evidence that the expression of these genes was significantly lower in type III SMA patients than in type II suggests that they may impact some clinical features, i.e., resulting in a different prognosis between the two groups, or the delayed onset, the different disabilities, or the response to therapies. Unfortunately, the small SMA type III group (five subjects) did not allow us to perform a solid statistical analysis to confirm this hypothesis; however, we believe that this result may be of some interest, and we hope that it will be validated by functional studies.

## 5. Conclusions

In this longitudinal, 12-month observation on adult SMA subjects treated with Risdiplam, the increase in SMN transcripts was verified, thus supporting the therapeutic efficiency of the molecule. Although exploratory, this evidence, together with its easy administration route (oral) and the very low occurrence of side effects, suggests that Risdiplam may be considered optional in SMA adults in whom the skeletal abnormalities related to the disease duration represent a severe limitation to intrathecal administration. Furthermore, at baseline, we observed several significant genetic differences between SMA type II and SMA type III subjects, which may be of interest in the clinical prospective evaluation as well as in searching for more focused therapeutic targets.

The absence of a contemporaneous healthy control group possibly limits the interpretation of some findings relative to physiological conditions. Specifically, we cannot determine whether the increased mitochondrial gene expression under Risdiplam reflects overactivation or compensation, nor whether the downregulation of autophagy- and lysosome-related pathways is beneficial or detrimental. However, the primary aim of this study was to assess longitudinal transcriptomic changes under Risdiplam treatment rather than case–control differences, and results should be interpreted within this framework.

## Figures and Tables

**Table 1:** Demographic and main clinical features of the recruited SMA subjects at baseline (T0) and after 12 months (T12).

Code	Sex	SMA Type	*SMN2* Copies	Age (Years)	Scoliosis	Spinal Fusion	Wheelchair	RULM (T0)	RULM (T12)	HFMS (T0)	HFMS (T12)
SMA13	F	type II	2	22	+	+	+	1	1	0	0
**SMA14**	F	type II	3	46	+	−	+	8	16	2	4
SMA15	M	type II	3	30	+	+	+	9	9	0	0
SMA16	F	type II	2	28	+	+	+	1	3	0	0
**SMA17**	F	type II	2	39	+	+	+	16	16	2	2
**SMA18**	M	type III	2	30	+	−	−	37	37	58	60
**SMA19**	F	type II	3	46	+	+	+	16	18	2	4
**SMA20**	M	type III	3	36	+	−	+	11	7	2	2
**SMA21**	F	type III	3	55	+	−	+	22	23	12	16
**SMA22**	M	type III	3	44	+	−	−	37	37	30	39
SMA23	F	type II	3	38	+	+	+	15	12	2	1
SMA24	M	type III	3	55	+	−	−	37	33	24	18
SMA25	F	type II	3	26	+	+	+	14	19	1	0
SMA26	F	type II	3	22	+	−	+	NA	NA	NA	NA
SMA27	M	type II	3	38	+	+	+	0	0	0	0
SMA28	F	type II	2	47	+	−	+	0	1	1	1

*Legend*: RULM = Revised Upper Limb Module; HFMS = Hammersmith Functional Motor Scale; in bold: patients with T12 sampling.

**Table 2:** Gene expression changes during Risdiplam. List of genetic regions with significantly different expression between T0 (16 SMA patients) and T12 (7 SMA subjects). From left to right, the columns contain the Gene ID (by Ensembl), the log_2_ fold change, and the *p*-adjusted values.

Gene_ID	log_2_FoldChange	*p*-Adjusted
ENSG00000249240	2.89	0.01305
ENSG00000309270	1.16	0.00775
ENSG00000228253_MT-ATP8	1.22	0.00274
ENSG00000228166_MTND1P11	0.65	0.00550
ENSG00000230225_MTND5P14	0.60	0.00230
ENSG00000250011_HMGB1P3	0.86	0.00149
ENSG00000298969	0.68	0.00601
ENSG00000251546_IGKV1D-39	−1.22	0.00550
ENSG00000260884_PCHILR	0.69	0.00601
ENSG00000228915_OR7E128P	0.75	0.01296
ENSG00000300700	0.74	0.00601
ENSG00000289888	0.75	0.00068
ENSG00000198899_MT-ATP6	1.04	0.00359
ENSG00000198786_MT-ND5	1.10	0.00290
ENSG00000198886_MT-ND4	0.95	0.00601
ENSG00000198695_MT-ND6	0.89	0.03738

**Table 3:** Changes in *SMN2* expression during Risdiplam. We mapped the RNA-seq reads on individual *SMN1/2* exon regions to evaluate the drug’s effect on correct transcript production. Although the counts were too low for formal statistical assessment, all patients show an increase in at least one of the exon portions of interest (fold change ≥ 1.5). This provides further confirmation of the drug’s effect on *SMN2* exon 7 splicing correction.

* Normalized Read Counts *	Exon 7 from *SMN1*	Exon 7 from *SMN2*	Exon 6–7 Junction from *SMN1*	Exon 6–7 Junction from *SMN2*	Exon 7–8 Junction	Ex. 6–7 and Ex. 7–8 from *SMN1*	Ex. 6–7 and Ex. 7–8 from *SMN2*
**SMA14_T0**	0	4.5	0	4.5	2.2	0	1.1
**SMA14_T12**	0	18.1	0	15.1	57.3	0	11.1
*** fold change ***		*** 4.0 ***		*** 3.4 ***	*** 25.6 ***		*** 9.9 ***
**SMA17_T0**	0	13.0	0	13.0	21.2	0	10.6
**SMA17_T12**	0	27.4	0	24.8	41.6	0	15.9
*** fold change ***		*** 2.1 ***		*** 1.9 ***	*** 2.0 ***		*** 1.5 ***
**SMA18_T0**	0	3.1	0	3.1	7.9	0	1.6
**SMA18_T12**	0	6.2	0	6.2	9.4	0	1.0
*** fold change ***		*** 2.0 ***		*** 2.0 ***	*** 1.2 ***		*** 0.7 ***
**SMA19_T0**	0	7.4	0	7.4	13.8	0	5.3
**SMA19_T12**	0	16.8	0	13.6	19.9	0	7.3
*** fold change ***		*** 2.3 ***		*** 1.8 ***	*** 1.4 ***		*** 1.4 ***
**SMA20_T0**	0	5.2	0	5.2	24.7	0	4.1
**SMA20_T12**	0	13.0	0	11.0	45.9	0	8.0
*** fold change ***		*** 2.5 ***		*** 2.1 ***	*** 1.9 ***		*** 1.9 ***
**SMA21_T0**	0	6.8	0	6.8	11.3	0	3.4
**SMA21_T12**	0	10.2	0	10.2	12.1	0	4.6
*** fold change ***		*** 1.5 ***		*** 1.5 ***	*** 1.1 ***		*** 1.4 ***
**SMA22_T0**	0	8.0	0	8.0	8.0	0	5.7
**SMA22_T12**	0	8.4	0	6.7	22.7	0	4.2
*** fold change ***		*** 1.1 ***		*** 0.8 ***	*** 2.8 ***		*** 0.7 ***

**Table 4:** Gene expression differences between SMA type II and SMA type III (at T0). List of genetic regions with significantly different expression between SMA type II patients (no. 11) and SMA type III patients (no. 5). From left to right, the columns contain the Gene ID (by Ensembl), the log_2_ fold change, the *p*-adjusted values, and the association with disorders of interest (and related references).

Gene_ID	log_2_FoldChange	*p*-Adjusted	References to Other Neuropsychiatric Diseases
ENSG00000149516_MS4A3	−1.55	0.00033	Candidate AD gene [36]
ENSG00000133112_TPT1	−1.31	0.00144	Neuronal differentiation [37]
ENSG00000147604_RPL7	−1.95	0.00715	Tauopathy [38]
ENSG00000145425_RPS3A	−1.81	0.01161	AD [39]
ENSG00000115128_SF3B6	−1.08	0.01456	Regulation of splicing factors, i.e., neural crest [40]
ENSG00000265681_RPL17	−1.45	0.01456	AD [41]
ENSG00000126860_EVI2A	−0.94	0.02012	Schizophrenia [42]
ENSG00000148908_RGS10	−1.07	0.02737	T-cell-mediated immunity in PD [43]
ENSG00000182903_ZNF721	−0.75	0.02737	Inflammation in PD [44]
ENSG00000122026_RPL21	−1.44	0.02984	T-cell immunity in AD [41]
ENSG00000123838_C4BPA	3.62	0.02991	AD [45]
ENSG00000182774_RPS17	−1.11	0.03029	AD [46]
ENSG00000198918_RPL39	−1.50	0.03137	Mitochondrial activity [47]
ENSG00000229117_RPL41	−1.46	0.03337	AD [48]
ENSG00000166710_B2M	−0.84	0.03916	AD and immunity [49]
ENSG00000039068_CDH1	−1.51	0.04366	Neuronal survival [50]
ENSG00000156482_RPL30	−0.97	0.04366	Schizophrenia [51]
ENSG00000109674_NEIL3	−1.83	0.04781	AD [52]
ENSG00000127920_GNG11	−1.12	0.04781	Cellular senescence [53]

## Data Availability

The raw data supporting the conclusions of this article will be made available by the authors upon request.

## Supplementary Materials

The following supporting information can be downloaded at: https://www.mdpi.com/article/10.3390/brainsci16060643/s1, Table S1: significant DE genes between T0 vs. T12 (PAIRED 7 subjects). Table S2: list of all the sequenced genetic regions. Table S3: most represented SMA modifier genes (completed list in Table S2). Table S4: list of genes belonging to SMN complex (out of Table S2). Figure S1: Structure of the enrichment analysis table. Term ID/Name indicates the biological process or pathway, together with the source database (e.g., GO, KEGG, Reactome etc.).
