# Supplementary material for: Longitudinal Transcriptomic Analysis Reveals Systemic Effects of Risdiplam in Adults with Spinal Muscular Atrophy
Source: Brain Sci. 2026 Jun 17;16(6):643. doi: 10.3390/brainsci16060643 (PMC13297622; doi:10.3390/brainsci16060643)
Supplement: Supplementary file 1 [file brainsci-16-00643-s001.zip › Supplementary Figure_S1.pdf]

***Figure S1.***

Structure of the enrichment analysis table. Term ID/Name indicates the biological process or pathway, together with the source database (e.g. GO, KEGG, Reactome etc.). Adjusted p-value refers to the multiple testing-corrected significance, with lower values (>0.05) indicating stronger enrichment. T denotes all genes annotated to a given term, Q represents the input gene list, and T ∩ Q indicates the overlap between these sets (input genes associated with the term). U corresponds to the total background set of genes used in the analysis (usually all genes in the selected genome).

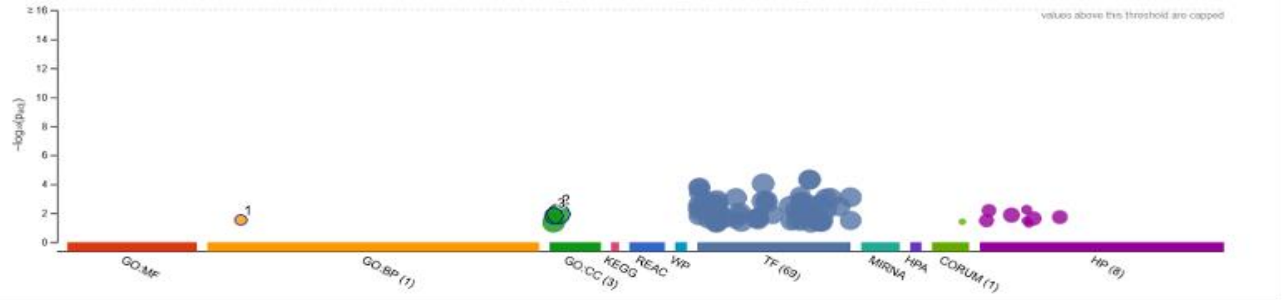


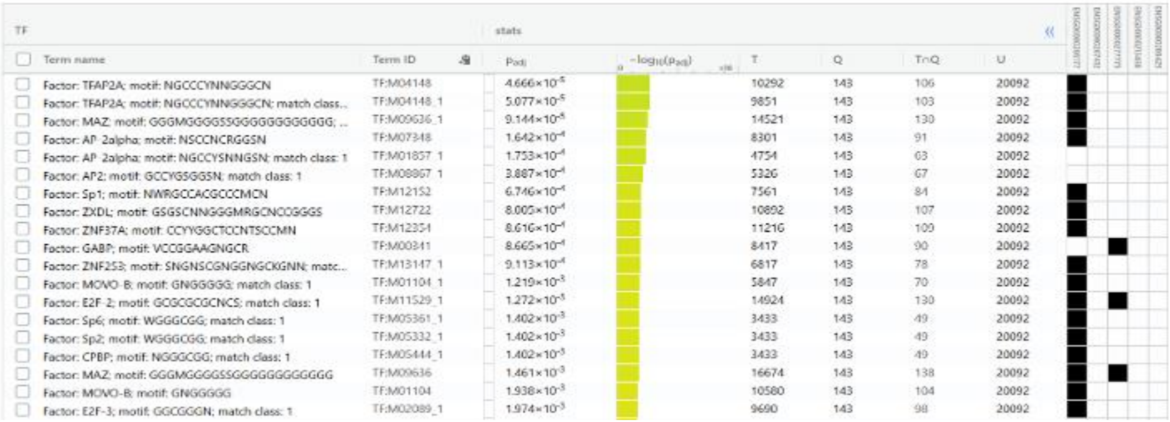

| TF | | stats | | | | | |
|---|---|---|---|---|---|---|---|
| Term name | Term ID | $P_{adj}$ | $-\log_{10}(P_{adj})$ | T | Q | T∩Q | U |
| Factor: TFAP2A; motif: NGCCCYNNGGGCN | TF:M04148 | $4.666\times10^{-5}$ | | 10292 | 143 | 106 | 20092 |
| Factor: TFAP2A; motif: NGCCCYNNGGGCN; match class... | TF:M04148_1 | $5.077\times10^{-5}$ | | 9851 | 143 | 103 | 20092 |
| Factor: MAZ; motif: GGGMGGGGSGGGGGGGGGGG; ... | TF:M09636_1 | $9.144\times10^{-5}$ | | 14521 | 143 | 130 | 20092 |
| Factor: AP-2alpha; motif: NSCCNCRGGSN | TF:M07348 | $1.642\times10^{-4}$ | | 8301 | 143 | 91 | 20092 |
| Factor: AP-2alpha; motif: NGCCYSNNGSN; match class: 1 | TF:M01857_1 | $1.753\times10^{-4}$ | | 4754 | 143 | 63 | 20092 |
| Factor: AP2; motif: GCCYGSGGSN; match class: 1 | TF:M08867_1 | $3.887\times10^{-4}$ | | 5326 | 143 | 67 | 20092 |
| Factor: Sp1; motif: NWRGCCACGCCCMCN | TF:M12152 | $6.746\times10^{-4}$ | | 7561 | 143 | 84 | 20092 |
| Factor: ZXDL; motif: GSGSCNNGGGMRGCNCCGGGS | TF:M12722 | $8.005\times10^{-4}$ | | 10892 | 143 | 107 | 20092 |
| Factor: ZNF37A; motif: CCYYGGCTCCNTSCCMN | TF:M12351 | $8.616\times10^{-4}$ | | 11216 | 143 | 109 | 20092 |
| Factor: GABP; motif: VCCGGAAGNGCR | TF:M00341 | $8.665\times10^{-4}$ | | 8417 | 143 | 90 | 20092 |
| Factor: ZNF253; motif: SNGNSCGNGGNGCKGNN; matc... | TF:M13147_1 | $9.113\times10^{-4}$ | | 6817 | 143 | 78 | 20092 |
| Factor: MOVO-B; motif: GNGGGGG; match class: 1 | TF:M01104_1 | $1.219\times10^{-3}$ | | 5847 | 143 | 70 | 20092 |
| Factor: E2F-2; motif: GCGCGCGCNCS; match class: 1 | TF:M11529_1 | $1.272\times10^{-3}$ | | 14924 | 143 | 130 | 20092 |
| Factor: Sp6; motif: WGGGCGG; match class: 1 | TF:M05361_1 | $1.402\times10^{-3}$ | | 3433 | 143 | 49 | 20092 |
| Factor: Sp2; motif: WGGGCGG; match class: 1 | TF:M05332_1 | $1.402\times10^{-3}$ | | 3433 | 143 | 49 | 20092 |
| Factor: CPBP; motif: NGGGCGG; match class: 1 | TF:M05444_1 | $1.402\times10^{-3}$ | | 3433 | 143 | 49 | 20092 |
| Factor: MAZ; motif: GGGMGGGGSGGGGGGGGGGG | TF:M09636 | $1.461\times10^{-3}$ | | 16674 | 143 | 138 | 20092 |
| Factor: MOVO-B; motif: GNGGGGG | TF:M01104 | $1.938\times10^{-3}$ | | 10580 | 143 | 104 | 20092 |
| Factor: E2F-3; motif: GGCGGGN; match class: 1 | TF:M02089_1 | $1.974\times10^{-3}$ | | 9690 | 143 | 98 | 20092 |
