# Supplementary material for: Longitudinal Transcriptomic Analysis Reveals Systemic Effects of Risdiplam in Adults with Spinal Muscular Atrophy
Source: Brain Sci. 2026 Jun 17;16(6):643. doi: 10.3390/brainsci16060643 (PMC13297622; doi:10.3390/brainsci16060643)
Supplement: Supplementary file 1 [file brainsci-16-00643-s001.zip › Supplementary Table_S1_S3_S4.pdf]

Table S1. significant DE genes between T0 vs. T12 (PAIRED 7 subjects)
List of genetic regions resulted expressed with significant difference between T0 and T12 (no.7 SMA subjects – PAIRED SAMPLES).

From left to right, the columns contain the Gene ID (by Ensembl), the log2Fold Change and the p-adjusted values.

Gene_ID	log_2-FoldChange	p-adjusted
ENSG00000249240	3.57	0.00000
ENSG00000309270	1.43	0.00000
ENSG00000228253_MT-ATP8	1.39	0.00000
ENSG00000228166_MTND1P11	0.87	0.00003
ENSG00000256357	0.85	0.00266
ENSG00000229325_ACAP2-IT1	1.17	0.01003
ENSG00000287086	1.00	0.01725
ENSG00000263606_CHORDC1P4	1.29	0.02193
ENSG00000144712_CAND2	0.96	0.02610
ENSG00000281571	-1.50	0.02610
ENSG00000257181	1.88	0.02623
ENSG00000229267_SNHG31	1.14	0.02961
ENSG00000290021	-0.67	0.02961
ENSG00000272933_TRIM8-DT	1.72	0.03577
ENSG00000280828	1.07	0.03577
ENSG00000290383	2.01	0.03797
ENSG00000229344_MTCO2P12	1.27	0.04541

Table S3. most represented SMA modifier genes (completed list in Table S2).

Changes (T12 *vs*. T0) resulted from the comparisons between SMA patients in paired within-subject comparison (7 vs. 7) and all subjects (7 vs 16). From left to right, the columns contain the Gene ID (by Ensembl), the log2Fold Change and the p-adjusted values.

	**Within-subjects (7 vs 7)**		**All SMA (7 vs 16)**	
**gene_id**	**log2_FoldChange**	**p-adjusted**	**log2_FoldChange**	**p-adjusted**
ENSG00000104490_NCALD	0.08	1.0000	-0.12	0.9994
ENSG00000110880_CORO1C	-0.06	1.0000	-0.02	0.9994
ENSG00000135486_HNRNPA1	0.29	0.4835	0.30	0.1233
ENSG00000101752_MIB1	0.07	1.0000	0.02	0.9994
ENSG00000124486_USP9X	-0.18	1.0000	-0.11	0.9964
ENSG00000130985_UBA1	-0.14	1.0000	-0.09	0.9994
ENSG00000104490_NCALD	0.08	1.0000	-0.12	0.9994
ENSG00000070831_CDC42	-0.09	1.0000	-0.06	0.9994
ENSG00000017427_IGF1	-1.99	NA	-2.15	NA
ENSG00000171791_BCL2	0.15	1.0000	0.11	0.9994
ENSG00000109917_ZPR1	0.15	1.0000	0.08	0.9994
ENSG00000116001_TIA1	0.02	1.0000	-0.02	0.9994
ENSG00000135486_HNRNPA1	0.29	0.4835	0.30	0.1233
ENSG00000115317_HTRA2	-0.02	1.0000	-0.02	0.9994

Table S4. list of genes belonging to SMN complex (out of Table S2).

Changes (T12 *vs*. T0) resulted from the comparisons between SMA patients in paired within-subject comparison (7 vs. 7) and all subjects (7 vs 16). From left to right, the columns contain the Gene ID (by Ensembl), the log2Fold Change and the p-adjusted values.

	paired (7 vs 7)		all (7 vs 16)	
**gene_id**	**log2_FoldChange**	**p-adjusted**	**log2_FoldChange**	**p-adjusted**
ENSG00000046647_GEMIN8	0.04	1.0000	-0.14	0.9963
ENSG00000082516_GEMIN5	0.02	1.0000	0.02	0.9994
ENSG00000092208_GEMIN2	0.12	1.0000	0.01	0.9994
ENSG00000142252_GEMIN7	-0.49	1.0000	-0.49	0.2588
ENSG00000152147_GEMIN6	0.01	1.0000	0.01	0.9994
ENSG00000179409_GEMIN4	-0.03	1.0000	-0.13	0.9840
ENSG00000259496_GEMIN8P1	0.42	1.0000	0.64	0.8000
ENSG00000267348_GEMIN7-AS1	0.22	1.0000	0.18	0.8852
ENSG00000023734_STRAP	0.05	1.0000	0.01	0.9994
ENSG00000111554_MDM1	0.17	1.0000	0.15	0.9174
ENSG00000135679_MDM2	-0.06	1.0000	-0.05	0.9994
ENSG00000198625_MDM4	-0.01	1.0000	-0.03	0.9994
ENSG00000274452_U2	0.48	NA	-0.33	NA
ENSG00000199805_RNU1-134P	-0.33	1.0000	-0.03	NA
ENSG00000200795_RNU4-1	0.21	1.0000	-0.23	0.9994
ENSG00000202538_RNU4-2	0.79	1.0000	0.49	0.9994
ENSG00000206588_RNU1-28P	-0.33	1.0000	-0.88	0.9856
ENSG00000206596_RNU1-27P	-0.31	1.0000	-0.07	0.9994
ENSG00000206652_RNU1-1	-0.33	1.0000	-0.88	0.9856
ENSG00000207005_RNU1-2	-0.31	1.0000	-0.07	0.9994
ENSG00000207389_RNU1-4	-0.31	1.0000	-0.07	0.9994
ENSG00000207513_RNU1-3	-0.33	1.0000	-0.88	0.9856
ENSG00000222328_RNU2-2P	0.09	1.0000	-0.07	0.9994
ENSG00000222973_RNU2-25P	0.28	1.0000	-0.10	0.9994
ENSG00000223336_RNU2-6P	0.32	1.0000	0.33	NA
ENSG00000252707_RNU11-2P	0.35	1.0000	0.41	0.9840
ENSG00000264229_RNU4ATAC	1.16	1.0000	0.54	0.9994
ENSG00000272359_RNU4-89P	0.89	1.0000	0.30	NA
ENSG00000199568_RNU5A-1	1.38	NA	0.19	NA

ENSG00000200156_RNU5B-1	1.49	NA	0.27	NA
ENSG00000200779_RNU6-105P	0.36	NA	0.85	NA
ENSG00000201616_RNU1-91P	1.14	NA	0.33	NA
ENSG00000221571_RNU6ATAC35P	0.03	NA	0.56	NA
ENSG00000221676_RNU6ATAC	0.86	NA	0.78	NA
ENSG00000222276_RNU2-33P	1.01	NA	0.41	NA
ENSG00000222389_RNU2-28P	-0.28	NA	-0.26	NA
ENSG00000222808_RNU4-47P	0.26	NA	0.32	NA
ENSG00000252118_RNU6ATAC39P	0.74	NA	0.64	NA
ENSG00000252391_RNU6-638P	0.93	NA	0.87	NA
ENSG00000276027_RNU12	1.33	NA	0.75	NA
ENSG00000100462_PRMT5	-0.01	1.0000	-0.15	0.9994
ENSG00000237054_PRMT5-AS1	0.09	1.0000	0.08	0.9994
ENSG00000257285_PRMT5-DT	-0.25	1.0000	-0.30	0.9143
ENSG00000164902_PHAX	0.16	1.0000	0.13	0.9840
ENSG00000082898_XPO1	-0.02	1.0000	-0.05	0.9994
ENSG00000132341_RAN	-0.05	1.0000	-0.13	0.9994
ENSG00000075624_ACTB	-0.18	1.0000	-0.15	0.9840
ENSG00000107290_SETX	-0.01	1.0000	0.04	0.9994
